# An Evaluation of the Postgraduate Physician Assistant/Associate and Nurse Practitioner Orthopedic Surgery Fellowship and Residency Websites in the United States

**DOI:** 10.7759/cureus.29875

**Published:** 2022-10-03

**Authors:** Vasco D Kidd

**Affiliations:** 1 Orthopaedic Surgery, University of California, Irvine, School of Medicine, Orange, USA

**Keywords:** website content, medical education, orthopedic surgery, advanced practice providers, transition-to-practice, fellowship, residency, nurse practitioner, physician associate, physician assistant

## Abstract

Background

Although considerable research on physician fellowship training program websites has demonstrated critical deficiencies in accessibility and content, there is no published study regarding the website content of physician assistant/associate (PA) and nurse practitioner (NP) orthopedic surgery postgraduate residency/fellowship programs in the United States. Therefore, this study aims to conduct a web-based content analysis to assess the available information on program websites and identify the potential areas for improvement.

Methods

The methodology for this study was replicated from prior research on graduate medical education (GME) websites. Twenty-two PA and joint PA/NP orthopedic surgery postgraduate residency/fellowship training program websites were assessed between August and September 2022. The criteria comprised 17 items related to postgraduate education and recruitment content. All program websites were evaluated for pertinent content relevant to prospective applicants applying to orthopedic surgery residency/fellowship programs. Descriptive statistics were performed.

Results

Out of the 22 PA and joint PA/NP orthopedic postgraduate websites evaluated, all had a functional website link. Only one orthopedic surgery postgraduate website met all 17 criteria. All program websites assessed contained program descriptions and contact information. Other information present on websites included program admission requirements 95% (21/22), salary/benefits 77% (17/22), interview requirement 73% (16/22), faculty listing 68% (15/22), journal club 59% (13/22), program objectives/goals 59% (13/22), and acceptance process 54% (12/22). Less than half of the program websites contained the following information: listing of current PA/NP postgraduate trainees, sample rotation schedule, orientation activities, curriculum, wellness/well-being strategy, graduation and research requirements, and link to program handbook. However, accredited PA orthopedic surgery program websites contained more content relevant to prospective applicants than non-accredited programs.

Conclusion

This is the first study to provide a detailed analysis of PA and joint PA/NP postgraduate orthopedic surgery residency/fellowship program websites. Not surprisingly, there is a wide variation in the available content on these websites. Study results provide further evidence to support the urgent need to improve program website content for prospective applicants.

## Introduction

Physician assistants/associates (PAs) and nurse practitioners (NPs) often referred to as advanced practice providers (APPs) have been a linchpin of the American Health Care system since the 1960s. PAs are educated as generalists in the medical model, whereas NPs are educated in at least one of six population foci [1,2]. The growth of PAs and NPs is partly motivated by industry changes including projected physician shortages, expanded access to care, an upward trend in value-based payment models, and improved profitability among hospitals and health systems [3,4]. Like physicians, PAs and NPs practice in all areas of medicine. While not required for state licensure or entry-level clinical practice, PA and NP postgraduate specialty training usually (12 months in length) is available in a variety of medical and surgical specialties [5-7]. According to a 2021 report, 5,851 PAs have completed some type of postgraduate training program [8]. However, the clinical and net economic benefits of completing a PA or NP residency/fellowship program remain a subject of debate.

Postgraduate specialty training opportunities are designed to provide greater depth in a specialty or subspecialty area not covered in an entry-level PA and NP education program. Additionally, newly minted PAs and NPs may find these programs attractive because of the opportunity to hone their clinical skills or acquire new ones. For example, Hart and Bowen surveyed several hundred licensed NPs and reported that 90% of them felt either “well prepared” or “somewhat prepared” for practice upon completion of their NP program and expressed interest in transition-to-practice opportunities through a formalized training program such as a residency [9]. Although, there are several descriptive cross-sectional studies describing programmatic functions and educational characteristics of PA and NP postgraduate fellowship/residency programs [7,10-13], the effect of PA and NP formalized specialty training on patient outcomes is unknown. Notwithstanding the fact that PA postgraduate residency/fellowship training has been around for nearly 50 years and NP residency training for almost two decades, some prospective applicants may be unaware of how to obtain information about these specialty training opportunities.

Currently, there is no national resident matching program or centralized application-processing service available to licensed PAs and NPs interested in pursuing postgraduate residency/fellowship training [10]. Therefore, prospective orthopedic surgery PA and NP applicants often use the internet as a source of information when researching and applying for these specialty training programs. Hence, the primary aim of this study is to perform a website content analysis of PA and joint PA/NP postgraduate orthopedic surgery residency/fellowship programs within the United States.

## Materials and methods

A comprehensive list of PA and joint PA/NP postgraduate orthopedic surgery residency/fellowship programs was compiled from the following sources: the Association of Post Graduate Advanced Practice Registered Nurse (APRN) Programs (APGAP), the Association of Postgraduate PA Programs (APPAP) websites, a Google search strategy, and from prior research on PA and NP postgraduate orthopedic surgery programs [6]. The Google search strategy was conducted using the search terms such as “physician assistant orthopedic surgery residency” and “nurse practitioner orthopedic surgery residency” to identify additional programs. Only programs with a functional website were included in this study. After a complete list of PA and joint PA/NP postgraduate orthopedic surgery residency/fellowship programs was finalized, a second Google search strategy using the program’s name was conducted to determine the accessibility of each program’s dedicated website (Table 1).

**Table 1:** Physician assistant and nurse practitioner postgraduate orthopedic surgery residency/fellowship programs in the United States

Postgraduate Programs (n = 22)
Duke University Physician Assistant Orthopedic Residency Program
Mayo Clinic's Orthopedic Sports Medicine Physician Assistant Fellowship
University of California San Francisco Orthopedic Surgery Physician Assistant Residency
University of California Davis Advance Practice Provider Fellowship
University of Rochester Medical Center Advanced Practice Provider Fellowship Program in Orthopedic Surgery
Wake Forest Orthopedic Surgery Physician Assistant Fellowship
The Medical College of Wisconsin Nurse Practitioner and Physician Assistant Postgraduate Orthopedic Fellowship
Ohio State Advanced Practice Provider Orthopedic Fellowship Postgraduate Program
Texas Children’s Hospital Orthopedic Surgery Physician Assistant Fellowship Program
Atlanta and Mercer University Physician Assistant Orthopedic Surgery Residency Program
Illinois Bone and Joint Institute Orthopedic Surgery Physician Assistant Residency
Carilion Clinic Orthopedic Surgery Nurse Practitioner and Physician Assistant Fellowship
Northwell Advanced Clinical Provider Fellowship in Orthopedics
U.S. Army/Baylor University Orthopedic Physician Assistant Residency
Arrowhead Regional Medical Center Orthopedic Surgery Fellowship Program
Indiana University Health-Postgraduate Advanced Practice Provider Training Program
New England Baptist Hospital Orthopedic Surgery Physician Assistant Fellowship
Riverside University Health System Medical Center Orthopedic Surgery Physician Assistant Fellowship Program
The Steadman Philippon Research Institute Sports Medicine Physician Assistant Fellowship Program
Navy Physician Assistant Graduate Training: Orthopedics
Direct Orthopedic Care Physician Assistant Residency Program: Choosing Orthopedics as a Career Specialty
Orlando Health Orthopedic Advanced Practice Provider Fellowship

Other external sources of information outside of the program’s dedicated website were not examined due to the potential for outdated and/or incomplete information. Each program's website home page was scanned along with content searches within each website to identify the presence of 17 criteria based on two categories. The postgraduate recruitment category included a listing of faculty and current fellows/residents, admission requirements, program description, acceptance process, salary/benefits, contact information, interview requirement, and program objectives/goals. The postgraduate education category included journal club, sample rotation schedule, orthopedic curriculum, orientation activities, graduation and research requirements, resident wellness/well-being, and hyperlink to program handbook. All 17 criteria were weighted appropriately. For example, when a content item was found on the program’s website homepage and/or subpages, it was awarded 1 point, but if absent, it was awarded 0 points. The rationale for including certain criteria elements in this study is based on the findings from previous research. For instance, in one study, medical students applying to surgical specialties indicated that fellowship acquisition, faculty information, application contact information, and resident wellness are the most important website content [14]. Another study of interventional radiology program applicants indicated that applicants were most interested in website content that contained didactics and information about facilities [15]. A study of pediatric orthopedic fellowship program websites found that information about research, affiliated hospital information, and rotations were the most prevalent education criteria on program websites [16]. Consistent with the prior research on orthopedic surgery residency websites, the data elements from all PA and joint PA/NP program websites were uniformly collected into an extraction grid template for analysis, which was modeled based on the one developed by Sherman et al., 2020 [17].

This study does not require institutional review board approval since the information assessed is from publicly available websites and there was no contact with residency/fellowship programs. 

## Results

The 22 PA and joint PA/NP postgraduate orthopedic surgery residency/fellowship programs are located in seven specific US census regions/divisions (Table 2).

**Table 2:** Demographic distribution of postgraduate orthopedic surgery residency/fellowship programs

United States Divisions/Region	Number of Postgraduate Programs
South Atlantic	6
East North Central	4
Pacific	4
West South Central	3
Middle Atlantic	2
Mountain	2
New England	1
West North Central	0
East South Central	0

There was variation in the content covered across websites. Out of the 22 PA and joint PA/NP orthopedic postgraduate websites evaluated, all had a functional website link. Only one orthopedic surgery postgraduate website met all 17 criteria. All program websites assessed contained program descriptions and contact information. Other information present on websites included program admission requirements 95% (21/22), salary/benefits 77% (17/22), interview requirement 73% (16/22), faculty listing 68% (15/22), journal club 59% (13/22), program objectives/goals 59% (13/22), and acceptance process 54% (12/22). Less than half of the program websites contained the following information: listing of current PA/NP postgraduate trainees, sample rotation schedule, orientation activities, curriculum, wellness/well-being strategy, graduation and research requirements, and link to program handbook (Table 3). Additionally, one program had as few as three of the 17 (18%) criteria identified on their website.

**Table 3:** Descriptive statistics for website content criteria

Program Website Content Criteria	Number of Websites, n% = 22
Program contact information	100% (22/22)
Program description	100% (22/22)
Admission requirements	95% (21/22)
Salary/benefits	77% (17/22)
Interview requirement	73% (16/22)
Faculty listing	68% (15/22)
Journal Club	59% (13/22)
Program objectives/goals	59% (13/22)
Acceptance process	54% (12/22)
Curriculum	45% (10/22)
Rotation schedule	32% (7/22)
Orientation activities	32% (7/22)
Research requirement	32% (7/22)
Residents' wellness/well-being strategy	27% (6/22)
Listing of current PA/NP fellows or residents	18% (4/22)
Graduation requirements from postgrad program	18% (4/22)
Program Handbook	14% (3/22)

However, 14% (3/22) accredited PA orthopedic surgery program websites contained more content criteria relevant to prospective applicants than non-accredited programs. Not surprising, given that external accreditation requires strict compliance with administrative and educational standards. Nevertheless, PA and NP postgraduate residency/fellowship specialty programs are not required to seek accreditation. Although not part of the original criteria, four websites (18%) included online video content, which offered a compelling view of postgraduate specialty training.

## Discussion

This is a rare study to examine the accessibility and presence of website content pertinent to PA and NP orthopedic surgery residency/fellowship applicants. Usability testing and quality assessment of the information provided on program websites were not conducted. While the results of this study indicate that the most prevalent information on program websites were program descriptions, contact information, salary/benefits, and interview requirement, there were substantial gaps in education and recruitment content. Information details about wellness, graduation requirements, sample rotation schedule, curriculum, link to program handbook, and listing of current residents/fellows were least readily available. These important findings coupled with existing research on GME programs highlight the value of enhancing program website content, which may improve orthopedic surgery recruitment efforts [17,18]. Moreover, research seems to suggest that website content and quality may influence an applicant’s program choice. For example, a study of emergency medicine applicants showed that 41% reported not applying to certain programs based on information from their websites, and slightly over one-third of plastic surgery applicants stated that program website quality influenced their decisions to interview at a program [19,20]. Another study showed that 40% of prospective applicants to an internal medicine residency program found websites most useful when preparing for their interviews [21].

A call to action

Our world today is undeniably digital, where data, people, and devices converge. Information is readily available and highly sought after by prospective applicants. Therefore, postgraduate programs should regularly audit their website content to ensure their online information is easily accessible, loads quickly, and is up to date. As an immediate step, orthopedic surgery postgraduate programs should clearly articulate on their website for prospective applicants the minimum graduation requirements and essential knowledge, skills, and competencies that must be satisfied to earn the certificate of completion from the program. Also, program websites should include a listing of current fellows/residents along with faculty photos and biography that present faculty members' education and research interests to prospective applicants. This is underscored by a retrospective analysis using website analytics of an accredited emergency medicine residency training program, which found that the most popular content accessed was the “people” section of the website, which includes photos of current residents and faculty [22].

Additionally, postgraduate programs should display an example of a weekly rotation and call schedule on their websites for prospective applicants to help codify training expectations. This is extremely important as some applicants prefer lifestyle controllability over traditional extended shifts as burnout rates are reportedly higher among those undergoing overnight call rotations [23]. Furthermore, a link to a program handbook may help to demystify didactic and clinical expectations, program policies and procedures, approval of travel and vacation requests, wellness/well-being programs, remediation, and other pertinent information that could potentially assist prospective orthopedic surgery applicants in making informed application and enrollment decisions. Lastly, due to the absence of research on PA and NP postgraduate specialty training websites, this novel study may serve as a guide for orthopedic surgery PA and joint PA/NP postgraduate programs to improve and prioritize their online content. Moreover, other PA and NP postgraduate specialty training programs may find this study useful in examining their websites for improvement. 

Limitations

This study is not without limitations. First, data elements were initially completed by a single researcher on two separate occasions, which may have led to some degree of bias. To reduce bias, an independent reviewer blinded to the results of the first review extracted data on 12 websites (54.5%) to analyze the online search results. Any data collection discrepancies led to the author and independent reviewer reexamining the specific website to resolve any errors. Additionally, this study was conducted on websites from a single specialty, thus limiting generalizability to other PA and NP postgraduate specialties. Moreover, the content analysis did not account for any program website updates or development of any new orthopedic surgery residency/fellowship programs that occurred after the review period. Finally, 17 content items were chosen for this study, but there may have been other content items that PA and NP applicants also find important when considering whether to pursue postgraduate training such as facilities information, housing/neighborhood information, postgraduate job prospects, diversity statement, moonlighting opportunities outside of the program, and loan deferment options. 

## Conclusions

Website recruitment and education content appear uneven across orthopedic surgery postgraduate programs. Given the variation in the online program information, there is an urgent need to optimize the program website content for prospective PA and NP orthopedic surgery applicants. Future research is needed to identify whether specific website content influences the decision of PA and NP applicants to apply to a postgraduate program. Moreover, whether prospective applicants search for residency/fellowship program information from online resources offered by APPAP or APGAP is an area of emerging interest. Also, an investigation into the quality, usability, and accessibility of PA and NP postgraduate residency/fellowship website content across all specialties is warranted.
